# Effects on applying micro-film case-based learning model in pediatrics education

**DOI:** 10.1186/s12909-020-02421-w

**Published:** 2020-12-09

**Authors:** Yuan Pan, Xiuqi Chen, Qiuwen Wei, Jinmin Zhao, Xun Chen

**Affiliations:** 1 Department of Graduate Administration, Guangxi Medical University, Nanning, 22 Shuangyong Road, Nanning, Guangxi People’s Republic of China; 2 Department of Humanity and Social Sciences, Guangxi Medical University, Nanning, Guangxi People’s Republic of China; 3 Department of Pediatrics, the First Affiliated Hospital of Guangxi Medical University, 6 Shuangyong Road, Nanning, Guangxi People’s Republic of China

**Keywords:** Case-based learning, Micro-film, Teaching method, Paediatrics

## Abstract

**Background:**

In view of the harsh reality Chinese paediatricians face, the challenge of paediatric education is about instilling not only knowledge and clinical skills but also resilience and beliefs. The aim of the study is to explore a more effective method than the traditional lecture-based learning (LBL) model for optimizing educational outcomes by establishing an innovative, comprehensive, case-based learning (CBL) model combined with the micro-film technique (MF + CBL). This approach has four important components: interests (attraction), knowledge application, competency, and scenario coping skills.

**Methods:**

Experimental research was conducted via a controlled parallel group study. The total sample of 104 senior-year students (Chinese) majoring in clinical medicine was randomly divided into two groups. The experimental group was exposed to the MF + CBL model and the control group to the LBL model. Overall, the results were assessed after an 8-week course via a student self-assessment questionnaire, a satisfaction survey and the final examination.

**Results:**

The experimental group generally performed better than the control group on the student self-assessment (P<0.05), satisfaction survey (P<0.05), and final examination (80.02 ± 3.77 vs 73.65 ± 3.69, *P* = 0.000). The open question at the end of the questionnaire revealed that a small number of students did not favour the MF + CBL model due to its time- and energy-consuming features.

**Conclusions:**

Compared with LBL, the MF + CBL model was an innovative teaching method that promoted more comprehensive quality development. It represents an alternative model for optimizing the capacity of future paediatric doctors.

## Background

The shortage of paediatricians in China is alarming. According to statistics published in *Pediatrics* in 2019, China has approximately 135 thousand paediatricians; these doctors face a severe service gap of 1 paediatrician per 2500 children (1:2500). The situation will worsen as the drop-out rate increases to approximately 12.6% [1]. Thus, paediatrics is trapped in an undesirable situation from the perspective of medical students.

Considering the harsh realities Chinese paediatricians face, such as lower salaries but heavier workloads, pressure deriving from the high expectations of patients’ family members, and greater occupational risks for medical disputes than other specialists [2], paediatric education is facing the challenge of cultivating a competent workforce with knowledge, resilience and beliefs [3].

Paediatrics is a course that can offer a first impression of the profession to paediatric medical students, playing a crucial role in both training talent and changing the dire situation in children’s health care services. Therefore, the mission of paediatrics is to attract talent, impart knowledge, develop competence and improve scenario coping to tackle the challenge posed by complex physical-psycho-social problems [4–6].

The rapid development of health care needs and the relative lag in comprehensive teaching modes have created higher requirements for the medical education system and greater challenges for medical educators. Previous studies have revealed the limitations of the traditional lecture-based learning (LBL) model with respect to problem-solving, collaborative learning, and lifelong learning strategies and have noted side effects such as decreased motivation and poor self-study, practical application and critical thinking abilities [7–9].

Contemporary medical education applies the problem-based learning (PBL) model and case-based learning (CBL) model as innovative teaching strategies; these approaches, which feature small-group, case-based discussion [10], have been proven to be highly effective in improving student/faculty satisfaction, encouraging lifelong learning and curiosity, and improving clinical and critical thinking and problem-solving ability [11]. However, the CBL model surpasses the PBL model in target orientation and effective in-depth learning through advance preparation and guiding clarification [12].

Although the CBL model is proven to be helpful in promoting professional competence by creating opportunities to handle medical case situations [13] aimed at facilitating knowledge assimilation and strengthening critical thinking [14], scenario teaching filled with humanities [15] and social medicine elements is insufficient [16, 17]. In addition, the number and diversity of cases is limited [18], which decreases students’ learning interest [19].

An innovative model blending the micro-film technique with the CBL model (MF + CBL) may solve the above problems by establishing a sustainable learning mechanism including four important elements of education: interests (attraction), knowledge application [20], competency, and scenario coping skills. Blended micro-film teaching models have been widely used in humanities and social sciences teaching. As an artistic teaching form, micro-film can be helpful for case creation by folding social factors, such as interpersonal interaction and a sense of values, into scenarios. It leverages the expressive advantages of a film while being much shorter, entertaining and interactive [21]. The time-efficient production cycle and low cost ensure the feasibility of collecting typical case videos in daily clinical work. Moreover, case creation with video fragments protects infants and children from the risk of repeating direct exposure.

Like the CBL model, the MF + CBL model unfolds a case, through the storytelling technique [22], within a specific socio-cultural context, allowing medical students to see things in a different way by seeking meaning through the application of knowledge and deepening their understanding of the wisdom of the adage “to cure sometimes, to relieve often, to comfort always.” Nevertheless, little research has been conducted on the application of the MF + CBL model in paediatrics teaching. Thus, this paper will demonstrate the effect of the MF + CBL model by comparing the traditional LBL model and innovative MF + CBL model with respect to students’ capacity development.

## Methods

### Participants

The study population comprised 104 undergraduates (Chinese) in their senior year studying clinical medicine at Guangxi Medical University, including 42 males and 62 females aged 25–27 years. The sample size was calculated by the sample size calculator [23]. By using the satisfaction degree in previous study [7] of the experimental group (92.5%), and of control group (70%), α = 0.05, powder = 80%, Sampling Ratio = 1:1, the effective sample size needed was at least 44 students per group. All the participants including teachers and students were all signed the Informed Consent, which required keeping confidential about course materials. All the students were divided into two parallel groups (52 students per group) by random sampling with a random number generator. The group exposed to the MF + CBL model was named the experimental group, while the group exposed to the LBL model was named the control group. There was no significant difference between the two groups in gender (*P* = 0.842), age (*P* = 0.253) or academic record (*P* = 0.347) (Appendix 1).

### Design

#### General curriculum arrangement

The textbook used was Pediatrics (Wang WP, 2018, People’s Medical Publishing House, Ninth Edition), based on which the teachers developed PowerPoints as supplements. Pneumonia, asthma, hyaline membrane disease and meconium aspiration syndrome were the 4 chapters chosen for this study, highlighting pathogenesis, diagnosis and treatment. Each chapter was covered in 6 class hours over 2 weeks, for a total of 24 class hours over 8 weeks.

#### Experimental group (MF + CBL model) design

Based on the task-oriented model, the whole experimental group was divided into four sub-groups according to the four chosen chapters. Each sub-group was responsible for making a micro-film with the help of the teacher, who performed the advanced work of taping case videos of children’s symptoms, such as pneumonia, fever, cough, wheezing, cyanosis and dyspnoea. The whole 8-week MF + CBL process was divided into 2 periods.

**Fig. 1:**
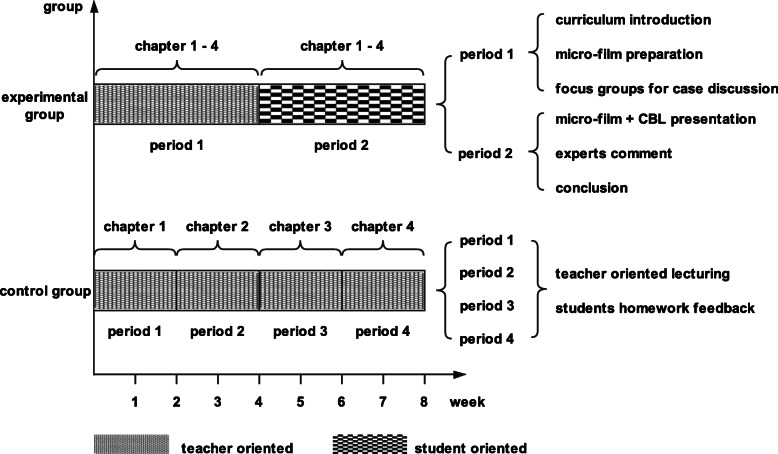
Course content and organisation

Period 1 was characterized by teacher-oriented CBL focus group discussion [24], while period 2 featured student-oriented micro-film presentations, followed by expert comments and conclusions (Fig. 1). In period 1, the teacher served as a leader to explain the experimental curriculum and requirements, clarified key knowledge points, demonstrated clinical observation and reasoning in wards, and assisted groups with information analysis in the focus group discussion. With respect to the micro-film making, students were required to present a case story containing both clinical factors, such as pathogenesis, diagnosis, and treatment, and related social medicine factors, such as poverty and humanism, sexual discrimination, and doctor-patient relationships. Students were encouraged to discuss these factors in a focus group under CBL structured questioning guidelines provided by the teacher [25].

In Period 2, students played a central role in the micro-film presentation. The teachers shifted to serving as organizers, arranging the class discussions and expert comments. The experts invited included clinicians, doctor-patient relation coordinators, social health management professors, members of the media and film industry.

The photographic equipment consisted of the iPhone X, 2017, Apple Inc., and the video editing software was Videoleap Pro, 2017 Lightricks Ltd., downloaded from the App Store.

#### Control group (LBL model) design

The whole teaching process was divided into 4 periods corresponding to the chosen chapters, which were covered in teacher-centred lectures with the help of PowerPoints and the necessary pictures and videos typically used, but no media such as micro-films were used. The teacher’s predominant role remained unchanged from beginning to end (Fig. 1).

Crucial potential confounding variables were controlled. In the 8-week teaching experiment, the experimental group (MF + CBL) and control group (LBL) used the same basic resources, including classroom sites and basic teaching materials (textbooks, PowerPoints, pictures, audio/video assistance, extra reading materials, etc.). In addition, all the teachers involved were at a similar level according to a comprehensive teaching evaluation and were trained in advance to avoid the introduction of subjective factors.

### Evaluation method

To realize a comprehensive evaluation, both process assessment and outcome assessment were applied in this research. A student self-assessment questionnaire and a satisfaction survey were used for process assessment (Appendix 2); in addition, the final closed-book examination scores were used for outcome assessment.

The student self-assessment questionnaire subjectively evaluated knowledge application, competency, and scenario coping skills by exploring students’ self-evaluation of their fundamental knowledge, clinical thinking, critical thinking, learning creativity, situation coping, and comprehensive capacity. The satisfaction survey mainly evaluated interests (attraction) by exploring students’ opinions about the teaching materials, methods and self-efficacy [26]. The final closed-book examination objectively evaluated the students’ command of fundamental knowledge and clinical comprehensive capacity by the last course of the 8-week experiment. In advance of the 8-week experiment, all the student participants were informed about the final closed-book examination, and there was no time for review. The final closed-book examination was designed and scored by all the teachers involved in the 8-week teaching experiment. The teachers signed confidentiality agreements, and the test papers were scored anonymously.

### Statistical analysis

All statistical analyses were performed with SPSS software (version 24.0), including the validity and reliability tests of all the measures. The measurement data were expressed in the form of^−^x ± s. Significance was assessed via an independent samples t-test. Categorical data were analysed by the chi-square test to compare teaching effects between the two groups. *P* < 0.05 was considered statistically significant.

## Results

### The validity and reliability of the measures

The validity and reliability of the three measures were tested, and the following results were obtained in Appendix 3.

### Comparison of the self-assessment results between the experimental group and the control group

The results of the self-assessment questionnaire revealed that the experimental group surpassed the control group in terms of fundamental knowledge (*P* = 0.010), clinical thinking (*P* = 0.038), situation coping (*P* = 0.000), critical thinking (P = 0.000), and learning creativity (P = 0.000) (Table 1). The open question at the end of the questionnaire revealed that a small number of students did not favour the MF + CBL model because it was more time consuming and energy consuming than self-study.

**Table 1:** Comparision of the self-assessment results between the experimental group and the control group

Group	MF + CBL (*n* = 52)		LBL (*n* = 52)		X^2^	P
Self - assessment	Yes	No	Yes	No		
Fundamental knowledge	40	12	29	23	4.306	0.038
Clinical thinking	43	9	30	22	6.618	0.010
Situation coping	50	2	18	34	40.827	0.000
Critical thinking	46	6	20	32	25.917	0.000
Learning creativity	40	12	18	34	17.190	0.000

Yes: satisfaction, No: dissatisfaction, MF + CBL: Case-based learning model with micro-film technique. LBL: Lecture-based learning

### Comparison of the final examination results between the experimental group and the control group

The final examination scores indicated that the experimental group had a higher aggregate score than the control group (80.02 ± 3.77 vs 73.65 ± 3.69, *P* = 0.000) and especially outperformed the control group in terms of fundamental knowledge (39.44 ± 2.65 vs 37.12 ± 2.24, P = 0.000) and case study (40.58 ± 1.97 vs 36.53 ± 2.54, P = 0.000) outcomes (Table 2).

**Table 2:** Comparison of the final examination results between the experimental group and the control group (scores,^−^x ± s)

Group	MF + CBL (*n* = 52)	LBL (*n* = 52)	χT value	*P* value
Fundamental knowledge	39.44 ± 2.65	37.12 ± 2.24	4.835	0.000
Case study	40.58 ± 1.97	36.53 ± 2.54	9.054	0.000
Total score	80.02 ± 3.77	73.65 ± 3.69	8.703	0.000

MF + CBL: Case-based learning model with micro-film technique. LBL: Lecture-based learning

### Comparison of the satisfaction survey results between the experimental group and the control group

The results of the satisfaction survey demonstrated that the experimental group was more satisfied than the control group in terms of lecture content (*P* = 0.010), teaching method (*P* = 0.029) and student self-efficacy (*P* = 0.025) (Table 3).

**Table 3:** Comparison of the satisfaction survey results between the experimental group and the control group

Group	MF + CBL (*n* = 52)			LBL (*n* = 52)			X^2^ value	*P* value
Contents	Yes	No	Degree	Yes	No	Degree		
Lecture content	46	6	88.46%	34	18	65.38%	6.554	0.010
Teaching method	43	9	82.69%	32	20	61.54%	4.782	0.029
Self - efficacy	44	8	84.62%	33	19	63.46%	5.002	0.025

Yes: satisfaction, No: dissatisfaction, Degree: degree of satisfaction, MF + CBL: Case-based learning model with micro-film technique. LBL: Lecture-based learning

## Discussion

**Fig. 2:**
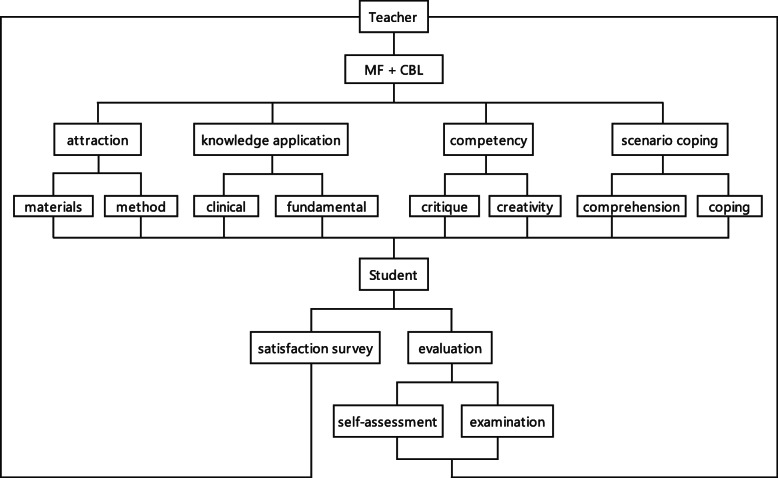
Teaching effect and evaluation

To meet the ever-changing challenges faced by medical education, this research aimed to establish a sustainable learning mechanism integrating interests (attraction), knowledge application, competency, and scenario coping skills. During this immersive learning process, students were required to intensively engage in case observation, analytical discussion and summarization, guided by the teacher. The teaching quality depended on the functional roles of the teacher and student in a loop of mutual reinforcement and was evaluated by designed assessments administered individually to both students and teachers (Fig. 2). Thus, the effect of the advanced teaching mode blending CBL with the micro-film technique is discussed below based on the results of all the measures.

First, the MF + CBL model was more attractive to students than the LBL model, as indicated by the results of the satisfaction survey on the three dimensions of the course content, the teaching method and student self-efficacy. Since the course content was the control variable, the teaching method and the learning process, as well as students’ feelings of enrichment, were more likely to influence the satisfaction level. The outcomes were probably due to the fact that, first, with the micro-film stories, the CBL model was updated to live-CBL or e-CBL [27], which was more vivid than lines in a textbook; second, the micro-films were an aesthetic fruit produced through medical students’ diligent interdisciplinary effort, and the presentation stimulated their inner potential with respect to teamwork, case reasoning and scaffolding for case presentations [28]; third, the CBL model, with its structured question technique, was more effective in guiding students to apply fundamental knowledge to practical clinic situations, narrowing the gap between knowledge and usage [29], which may have increased self-efficacy and thereby motivated learning initiative and creativity [30].

Second, MF + CBL model may be more effective than the LBL model with respect to knowledge application, which can be proved by the higher scores in terms of fundamental knowledge and case analysis in the experimental group (Table 2). The higher average score might reflect better capacity of memorizing, understanding and clinical comprehensive capacity. Since traditional LBL model was characterized by its teacher-centred efficiency in a cramming system, where students could only seize fragments of knowledge by rote [31], whereas the MF + CBL model increased students’ automatic processing by providing abundant opportunities for experiential practice [32]. In addition, the micro-film told a case story based on the development of an illness, which followed the natural laws of medical practice and knowledge assimilation, as well as encouraged the study of cases in context, within which students could realize knowledge application through the mutual reinforcement of learning and practice. However, the KMO value (0.500) of the closed-book examination, make Cronbach’s alpha (0.866) not fully reliable. The likely reasons may be the test paper contained only two parts, objective questions for fundamental knowledge and subjective questions for case analysis, which could not show a full picture of student’s capability. In further research, we will used more mature test papers or other measurements to testify the result.

Third, the MF + CBL model more effectively developed students’ learning capacity than the LBL model. Learning capacity can be evaluated by assessing critical thinking and learning creativity [31]. Requiring vast stores of knowledge, mastery of complex clinical situations and the incorporation of frontier learning, medical education not only focuses on the right answer but also on the right way through cultivating critical thinking based on a solid foundation of knowledge. Learning creativity can also help students persist in learning by motivating their thinking and inquiry. The reasons that the experimental group had higher “critical thinking” and “learning creativity” scores might be as follows: first, micro-film is a video expression technique that allowed information exploration. Students in the experimental group were encouraged to scan the available information for, for instance, important yet subtle clues, enhancing visual cue interpretation [32], or deviant behaviours obscured by dramatic plots, helping to trigger critical thinking. Second, the MF + CBL model was a creative learning method that could be assimilated by students as they improved their practical, medical humanities and evidence-based medicine competencies. Teaching did not end at the time of knowledge assimilation; instead, it continued in the construction of the student’s learning model. Individuals established their learning model by acquisition [31] and further influenced others.

Fourth, the MF + CBL model better promoted students’ situational understanding and coping than the LBL model. The social issues related to the patient or illness reflected in the MF + CBL model can expand students’ case discussions to the bio-psycho-social system of the patient [33]. Themes of social medical triggers, values affecting health service offerings, doctor-patient relationships, etc., can be revealed through structured guiding questions from the teacher in the MF + CBL focus group. Such questions may include “How can we design a family rehabilitation plan for the patient?”, “What are the risk factors for doctor-patient relationships in paediatrics? How can this relationship be improved?”, and “In the medical field, how can children with special needs be helped in a sustainable way?”. Thus, the learning procedure was also the gateway to practicing in scenarios [22]. Therefore, students were required to bring not only questions but also solutions to the case scenario. In addition, during the shooting process, students needed to interview hospital staff, patients and their families, and media staff, which deepened their comprehension of clinical practice and the dilemmas and social stress encountered in medical care, resulting in the improvement of their situational coping skills.

In reality, the MF + CBL model had limitations as a demanding teaching method for both students and teachers in terms of students’ willingness to engage in self-study, creative thinking, and expression and teachers’ capacity to handle innovative techniques and group discussion leading and conclusion [34]. In addition, initial planning and extracurricular activities were important parts of the MF + CBL model, and some students—in the experimental group—expressed dissatisfaction with this innovative trial. We do not deny that cramming could lead to a better grade on an exam in the current Chinese education system, since the way the student obtains knowledge is not inherent but acquired based on the learning context or environmental influence. Nevertheless, the seemingly superior efficiency of imparting knowledge through lectures was outweighed by the disadvantages in terms of lack of critical thinking, which might limit students’ capacity development to the path of mechanical memorization. The MF + CBL model was comparatively time-consuming if students sought to perform well but was useful in developing a deep approach to learning in students [31], which changed the target of learning from scores to strength.

### Limitations

This study has several limitations. First, the teachers volunteered recruited in LBL or CBL group, the subjective influence from teachers could not be denied, though all the teachers involved in this study were appraised as above average prior to study implementation. Second, the study sample was recruited from within one institution, and the process took place over only 8 weeks covering 4 chapters, which might not be a broad enough or long enough scope to observe a stable change in medical students’ educational outcomes. Third, the test paper design of closed book examination was imperfect reflected by the result of KMO value.

## Conclusions

The results indicated that the MF + CBL model had greater efficacy than the traditional LBL model. The MF + CBL model was preferred and broadly beneficial to both teachers and students in the 8-week teaching experiment. The ever-changing demands of the health care system have pushed doctors to a higher level of capacity, requiring deeper thinking and critical analysis during unforeseen and unfamiliar situations or in research. It is timely to explore further how educators can improve and alter clinical teaching methods with the MF + CBL model to optimize students’ learning effectively and to encourage a shift towards capacity building. Our findings call for long-term research covering the university educational and hospital clinical practice periods on the effect of curricular interventions on paediatrics to guide teaching innovation in medical education.

## Supplementary Information

**Additional file 1: Appendix 1.** Demographic data.

**Additional file 2: Appendix 2.** Questionnaire for self-assessment and satisfaction survey.

**Additional file 3: Appendix 3.** Reliability and validity analysis results of three test methods.

**Additional file 4: Appendix 4.** GUANGXI MEDICAL UNIVERSITY ETHICAL REVIEW COMMITTEE Approval Notice.

## Data Availability

All data generated or analyzed during this study are included in this published article and its supplementary information files.

## Abbreviations

CBL: Case-based learning
LBL: Lecture-based learning
MF + CBL: Case-based learning model with micro-film technique
