# Supplementary material for: Effects on applying micro-film case-based learning model in pediatrics education
Source: BMC Med Educ. 2020 Dec 9;20:500. doi: 10.1186/s12909-020-02421-w (PMC7727213; doi:10.1186/s12909-020-02421-w)
Supplement: Supplementary file 1 — Additional file 1: Appendix 1. Demographic data. [file 12909_2020_2421_MOESM1_ESM.doc]

Appendix 1. Demographic data

|  | Male | Female | Age / Year (x ± s ) | Academic record* (x ± s) |
| --- | --- | --- | --- | --- |
| MF + CBL (n = 52) | 22 | 30 | 25.85 ± 0.61 | 77.92 ± 5.41 |
| LBL (n = 52) | 20 | 32 | 25.96 ± 0.39 | 78.90 ± 5.16 |
| T / X^2^ value | 0.040^a^ | | -1.150^b^ | -0.946^c^ |
| P value | 0.842 | | 0.253 | 0.347 |

MF + CBL: Case-based learning model with micro-film technique. LBL: Lecture-based learning,

^a^: X^2^ value,

^b, c^: T value,

*: referenced by individual student’s average scores of main course last year.
