# Supplementary material for: Effects on applying micro-film case-based learning model in pediatrics education
Source: BMC Med Educ. 2020 Dec 9;20:500. doi: 10.1186/s12909-020-02421-w (PMC7727213; doi:10.1186/s12909-020-02421-w)
Supplement: Supplementary file 2 — Additional file 2: Appendix 2. Questionnaire for self-assessment and satisfaction survey. [file 12909_2020_2421_MOESM2_ESM.doc]

Appendix 2: Student self-assessment questionnaire and satisfaction survey

| Student self-assessment questionnaire | | |
| --- | --- | --- |
| 1. Do you agree that this 8-week course can solid your fundamental knowledge about pediatrics theories? | Yes | No |
| 1. Do you agree that this 8-week course can improve your clinical thinking when you facing a real case? | Yes | No |
| 1. Do you agree that this 8-week course can help you better coping with and well handling complex situations when facing patients and their [statutory guardian](javascript:;)s? | Yes | No |
| 1. Do you agree that this 8-week course can help you in developing critical thinking? | Yes | No |
| 1. Do you agree that after this 8-week course you can creatively try innovation methods in future learning, which you would be pleased to introduce to others? | Yes | No |
| Satisfaction survey | | |
| 1. Are you satisfied with this 8-week course contents, including all the materials teacher used? | Yes | No |
| 1. Are you satisfied with the teacher’s teaching method during this 8-week course? | Yes | No |
| 1. Are you satisfied with your overall performance during this 8-week course? | Yes | No |

Yes: satisfaction, No: dissatisfaction
