# Supplementary material for: Effects on applying micro-film case-based learning model in pediatrics education
Source: BMC Med Educ. 2020 Dec 9;20:500. doi: 10.1186/s12909-020-02421-w (PMC7727213; doi:10.1186/s12909-020-02421-w)
Supplement: Supplementary file 3 — Additional file 3: Appendix 3. Reliability and validity analysis results of three test methods. [file 12909_2020_2421_MOESM3_ESM.docx]

Appendix 3. Reliability and validity analysis results of three test methods

| Testing methods | Reliability analysis | |  | Validity analysis |
| --- | --- | --- | --- | --- |
|  | Cronbach's alpha | Cronbach's alpha based on standardized items |  | Kaiser-Meyer-Olkin measure of sampling adequacy |
| 1. Self-assessment questionnaire | 0.939 | 0.939 |  | 0.740 |
| 2. Satisfaction survey | 0.971 | 0.972 |  | 0.730 |
| 3. Final closed-book examination | 0.866 | 0.884 |  | 0.500 |
