# Supplementary material for: Effects on applying micro-film case-based learning model in pediatrics education
Source: BMC Med Educ. 2020 Dec 9;20:500. doi: 10.1186/s12909-020-02421-w (PMC7727213; doi:10.1186/s12909-020-02421-w)
Supplement: Supplementary file 4 — Additional file 4: Appendix 4. GUANGXI MEDICAL UNIVERSITY ETHICAL REVIEW COMMITTEE Approval Notice. [file 12909_2020_2421_MOESM4_ESM.pdf]

GUANGXI MEDICAL UNIVERSITY
ETHICAL REVIEW COMMITTEE
Approval Notice

Approval Number: 2019 (Edu-R-E-001)

Title: Effects on applying Micro-Film Case-Based Learning model in pediatrics education

Research Contents: The study population comprised 104 undergraduates (Chinese) in the senior year from the major of clinical medicine of Guangxi Medical University, including 42 male and 62 female, age from 25-27 year-old. All the participants were divided into two parallel groups (52 students per group) by random sampling with a random number generator. The experimental group will be intervened by Micro-Film Case Base Learning model model, while the control group by Lecture Based Learning model in the Pediatrics subject on 4 chapters: Pneumonia, Asthma, Hyaline membrane disease and Meconium aspiration syndrome during 8 weeks, in total 24 class hours. The learning effects of both group will be assessed by Student self-assessment questionnaire, Satisfaction Survey and final closed-book examination. All the cases and video materials used in course teaching are informed and obtained patients' statutory guardians' consent for medical educational usage. All student-participants are voluntary and signed declaration of informed consent before participating in the study. The micro-film making procedure strictly followed the medical ethics of patient-priority, respect and impartial. Infant model (KM/TY4, Shanghai Kangmu Science and Technology Co. Ltd, China) will be used in necessary extra video shooting to avoid any harmfulness to patients.

Applicant: Pan Yuan

Application Department: Information and Administration College

Date of Application: March 1st, 2019

Date of Approval: March 6th, 2019

Conclusion: This project fully considered and protected the rights and interests of the study objects. It meets the criteria of Ethical Review Committee. The Medical Ethics Committee of Guangxi Medical University has approved the protocol.

Ethical Review Committee
of Guangxi Medical University
March 6th, 2019
